# Correlation between Maternal Systemic Inflammatory Indicators before Delivery and Congenital Pneumonia in Newborns: A Case–Control Study

**DOI:** 10.3390/children11080985

**Published:** 2024-08-14

**Authors:** Tianping Bao, Bingrui Yu, Yu Chen, Yuan Zhang, Huaiping Cheng, Zhaofang Tian

**Affiliations:** Department of Neonatology, The Affiliated Huaian No.1 People’s Hospital of Nanjing Medical University, Huai’an 223300, China; hayybtp@njmu.edu.cn (T.B.); yubingrui@stu.njmu.edu.cn (B.Y.); ychen.med@stu.njmu.edu.cn (Y.C.); zhangyuan123@njmu.edu.cn (Y.Z.); hayychhp@njmu.edu.cn (H.C.)

**Keywords:** systemic inflammatory indicators, congenital pneumonia, full-term

## Abstract

Background: Congenital pneumonia is a common respiratory disease in newborns, often influenced by obstetric factors. Clinical diagnosis can be delayed, prompting interest in using systemic inflammatory indicators to predict various diseases. Objective: Our aim was to evaluate the predictive value of maternal systemic inflammatory indicators before delivery for congenital pneumonia in newborns. Methods: This retrospective study included full-term, singleton infants born at the Affiliated Huaian No. 1 People’s Hospital of Nanjing Medical University between January 2017 and December 2022. Infants admitted to the neonatal department within 48 h of birth were divided into two groups: 46 with clinical congenital pneumonia (the observation group) and 65 without congenital pneumonia (the control group). Maternal peripheral blood, complete blood cell count, and general condition within 72 h before delivery, as well as neonatal admission data were recorded. Systemic inflammatory indicators were assessed. Receiver operating characteristic curves were used to evaluate the predictive value of the maternal systemic inflammatory indicators before delivery for congenital pneumonia. A prediction model for neonatal pneumonia was constructed by combining systemic inflammatory indicators before delivery with logistic regression. The association between this prediction model and the prognosis of neonatal congenital pneumonia was examined. Results: Maternal systemic inflammatory indicators before delivery as predictive markers for congenital pneumonia and the regression model jointly constructed by NLR, MLR, SII, SIRI, and PIV before delivery predicted the occurrence of congenital pneumonia better. Maternal systemic inflammatory indicators correlated with the severity of congenital pneumonia in neonates. Conclusions: Maternal systemic inflammatory indicators before delivery have predictive value for congenital pneumonia in neonates, aiding early identification and treatment.

## 1. Introduction

Congenital pneumonia poses a significant threat during the neonatal period, often stemming from infectious agents transmitted via amniotic fluid or hematogenous routes, leading to diverse pathological changes. In severe cases, it can progress to septic shock and may be accompanied by persistent pulmonary hypertension. Its incidence is approximately 1% in full-term newborns and rises to 10% in premature infants, making it a leading cause of early neonatal mortality [1,2]. Given its association with obstetric factors, it is also known as intrauterine infectious pneumonia. Early diagnosis and treatment are crucial for preventing mortality and morbidity.

Currently, there are no universally accepted diagnostic criteria for congenital pneumonia. Diagnosis relies heavily on clinical symptoms and chest X-ray findings in newborns. However, the lack of specific clinical manifestations often delays diagnosis, and early chest X-rays pose radiation risks while not definitively distinguishing pneumonia from other conditions, complicating clinical assessment and potentially leading to missed diagnoses [3]. Lung ultrasound, known for its high sensitivity and specificity in diagnosing neonatal lung diseases, has been widely used in neonatal intensive care units (NICUs). However, it faces challenges in distinguishing among various neonatal respiratory conditions, such as respiratory distress syndrome, neonatal transient tachypnea, meconium aspiration syndrome, and neonatal pneumonia [4]. Consequently, there is ongoing interest in identifying clinical markers for diagnosing congenital pneumonia, a focal point in current clinical research.

Studies indicate promising avenues for the early diagnosis of congenital pneumonia. For instance, examining tracheal aspirates within eight hours after birth has shown utility in early diagnosis [3]. Furthermore, neonates with congenital pneumonia exhibited significantly lower serum vitamin D levels compared to a control group [2], suggesting fetal vitamin D deficiency as a potential predisposing factor. Additionally, serum cathelicidin antimicrobial peptide levels are significantly increased in neonates with congenital pneumonia [5]. Moreover, procalcitonin has emerged as another potential early marker for diagnosing congenital pneumonia [6].

Systemic inflammation indicators play a crucial role in predicting and diagnosing various clinical diseases. For example, the neutrophil-to-lymphocyte ratio (NLR) has demonstrated strong predictive and prognostic capabilities in infectious diseases [7]. In very low-birth-weight premature infants with early-onset sepsis, NLR, monocyte-to-lymphocyte ratio (MLR), and systemic inflammation response index (SIRI) values were all significantly elevated compared to the control group, highlighting the predictive value of SIRI for early-onset sepsis [8]. Previous studies also indicated that systemic inflammation indicators like neutrophil count (N), NLR, and SIRI can effectively predict bronchopulmonary dysplasia (BPD) in premature infants, aiding in their clinical management [9].

Maternal hematology indicators are closely associated with maternal and neonatal perinatal disease. Mothers diagnosed with threatened preterm birth within one week showed significantly higher median NLR and platelet-to-lymphocyte ratio (PLR), both of which exhibited high sensitivity and specificity in predicting spontaneous preterm birth [10]. Additionally, a case–control study by Levy et al. highlighted that maternal hematological biomarkers in early pregnancy are associated with neonates who are small for gestational age (SGA). Elevated NLR values in early pregnancy were notably higher in mothers of SGA infants, with significantly increased NLR levels observed in severe cases of SGA, suggesting NLR as a potentially effective early marker for predicting SGA [11]. Despite advancements, research on the relationship between maternal systemic inflammation indicators before delivery and neonatal congenital pneumonia remains limited.

This retrospective cohort study aims to analyze this relationship, assessing the predictive value of maternal systemic inflammatory indicators for congenital pneumonia in newborns.

## 2. Material and Methods

### 2.1. Study Participants

Single-term infants delivered at the obstetrics department of the Affiliated Huaian No. 1 People’s Hospital of Nanjing Medical University between January 2017 and December 2022, who were admitted to the neonatal department within 48 h of birth, were included in this study. Participants were divided into two groups: 46 newborns diagnosed with congenital pneumonia formed the observation group, while 65 newborns without congenital pneumonia during the same period comprised the control group. The general conditions of mothers and infants, along with hematological parameters, were recorded. Demographic data were collected from hospital medical records. Inclusion criteria included (1) clinical diagnosis of neonatal congenital pneumonia as per established criteria [5], characterized by symptoms like tachypnea, intercostal or subcostal retractions, groaning, and specific chest radiograph findings shortly after birth, such as nodular or coarse patchy infiltration, diffuse haziness or granularity, air bronchograms, and lobar or segmental consolidation, with all chest X-rays reviewed by a radiologist; (2) admission age ≤ 48 h; (3) full-term birth; (4) singleton birth; and (5) complete clinical data. Exclusion criteria included (1) gestational age < 37 or >42 weeks; (2) twin or multiple births; (3) severe congenital malformations, hyaline membrane disease, or congenital heart disease; (4) non-delivery or non-admission to the neonatology department of our hospital; and (5) incomplete clinical data. This study was approved by the Medical Ethics Committee of our hospital (ethical approval No.: KY-2024-194-01). Informed consent was obtained from pregnant women and their families before specimen collection.

### 2.2. Methods

Peripheral venous blood samples were collected within 72 h before delivery and within 24 h after admission to the neonatology department using ethylenediaminetetraacetic acid tubes. Complete blood counts were analyzed using a SYSMEXXN-9000 automated blood cell analyzer (KHB, Shanghai, China). The following parameters were recorded: N (10^9^/L), platelet count (P; 10^9^/L), monocyte count (M; 10^9^/L), and lymphocyte count (L; 109/L). Systemic inflammatory indicators such as NLR, PLR, MLR, systemic immune inflammation index (SII), SIRI and pan-immune-inflammation value (PIV) were calculated as follows: NLR = N/L, PLR = P/L, MLR = M/L, SII = N × P/L, SIRI = N × M/L, and PIV = P × N × M/L.

### 2.3. Statistical Analysis

Data analysis was conducted using IBM SPSS Statistical Software for Windows (version 27.0; IBM Corp., Armonk, NY, USA). For normally distributed measurement data, results are presented as mean ± standard deviation (x̄ ± s), and between-group comparisons were assessed using the *t*-test. Non-normally distributed measurement data are expressed as median (interquartile range) [M (P25, P75)], and between-group comparisons were performed using the Mann–Whitney U test. Categorical data are expressed as percentages, and between-group comparisons were conducted using the chi-square test (χ^2^ test). The diagnostic value was evaluated using receiver operating characteristic (ROC) curves, with the cutoff value defined as the index yielding the closest approximation. A *p*-value of <0.05 was considered statistically significant.

## 3. Results

### 3.1. Clinical Condition of Mothers and Neonates

A significant difference was observed between the two groups of neonates in terms of a low one-minute Apgar score (*p* < 0.05). No significant differences were observed between the two groups of neonates in terms of birth weight, sex, gestational age, and other aspects (Table 1).

### 3.2. Systemic Inflammatory Indicators of the Mothers before Delivery

The mothers in the observation group exhibited significantly higher NLR, MLR, SII, SIRI, and PIV levels compared to those in the control group (*p* < 0.05), while no statistical difference was observed in PLR between the two groups (Table 2).

### 3.3. Comparison of Systemic Inflammatory Indicators of the Neonates between the Two Groups

No statistically significant differences were found in the NLR, PLR, MLR, SII, SIRI, or PIV between the pneumonia and control groups (Table 3).

### 3.4. The Effect of Systemic Inflammatory Indicators of the Mothers before Delivery on Predicting Congenital Pneumonia in the Neonates

**Figure 1:**
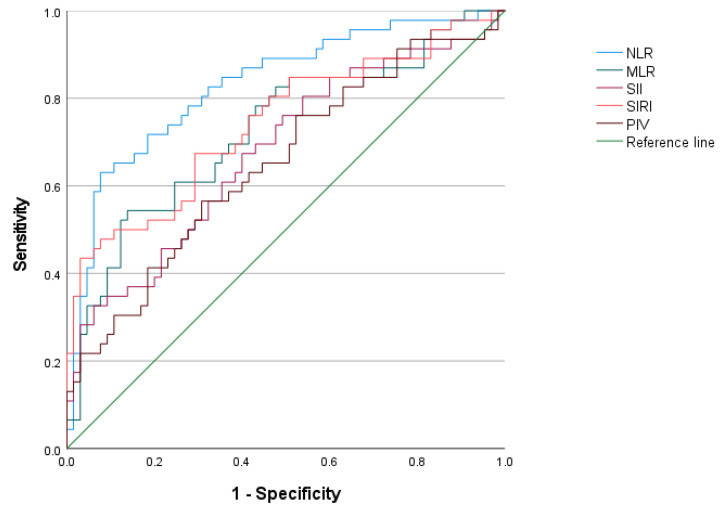
ROC curves for evaluating the effect of systemic inflammatory indicators of the mothers before delivery on the diagnosis of congenital pneumonia in the neonates. ROC, receiver operating characteristic curve; NLR, neutrophil-to-lymphocyte ratio; MLR, monocyte-to-lymphocyte ratio; SII, systemic immune-inflammation index; SIRI, systemic inflammation response index; PIV, pan-immune-inflammation value.

We compared the ROC curves of the systemic inflammatory indicators of the mothers before delivery and observed that prepartum NLR had the highest predictive power for congenital pneumonia in newborns, with an AUC of 0.830, the optimal cutoff value at this time was 4.368, the sensitivity was 63.0%, and the specificity was 92.3% (Figure 1, Table 4).

### 3.5. Constructing a Predictive Model for Neonatal Pneumonia Using a Combination of Indicators and Logistic Regression

**Figure 2:**
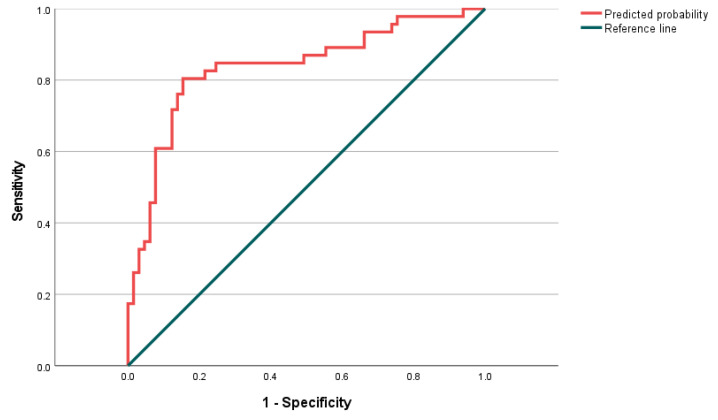
ROC curve for evaluating the effect of NLR, combined with MLR, SII, SIRI, PIV of the mothers before delivery on the diagnosis of congenital pneumonia in the neonates. ROC, receiver operating characteristic curve; NLR, neutrophil-to-lymphocyte ratio; MLR, monocyte-to-lymphocyte ratio; SII, systemic immune-inflammation index; SIRI, systemic inflammation response index; PIV, pan-immune-inflammation value.

A prediction model for neonatal pneumonia was constructed by combining five maternal systemic inflammatory indicators of mothers before delivery (NLR, MLR, SII, SIRI, and PIV) with logistic regression (Figure 2), with an AUC of 0.837 (*p* < 0.001) and a 95% CI of 0.756–0.917. The maximum value of Youden’s J statistic was 0.65; furthermore, the optimal cutoff value at this time was 0.369, the sensitivity was 80.4%, and the specificity was 84.6%.

### 3.6. Correlation between Maternal Systemic Inflammatory Indicators before Delivery and the Severity of Congenital Pneumonia in Newborns

The predicted probability was divided into two groups: predicted probability < 0.369 and predicted probability > 0.369. Children with a high predicted probability have longer hospital stays, as well as a higher likelihood of antibiotic use and oxygen demand (*p* < 0.05, Table 5).

## 4. Discussion

This retrospective study analyzed 46 full-term neonates with congenital pneumonia and 65 full-term control neonates. We found a low one-minute Apgar score in the pneumonia group compared to the control group, consistent with the findings of Taiorazova et al. [2]. Furthermore, the maternal systemic inflammatory indicators before delivery were able to predict congenital pneumonia. Areas under ROC curves for prepartum NLR, MLR, SII, SIRI, and PIV were 0.830, 0.727, 0.677, 0.742, and 0.651, respectively. The sensitivity was 0.630, 0.543, 0.674, 0.435, and 0.565, respectively, and the specificity was 0.923, 0.862, 0.600, 0.969, and 0.692. The AUC of the neonatal pneumonia prediction model constructed using logistic regression was 0.837, the sensitivity was 80.4%, and the specificity was 84.6%, which were better than the prediction using any of the five maternal systemic inflammatory indicators of mothers before delivery alone.

Maternal blood indicators play a crucial role in predicting neonatal diseases. For instance, SII and SIRI values have been utilized alongside clinical findings to predict adverse perinatal outcomes in mothers with coronavirus disease 2019 [12]. In mothers with preeclampsia, there exists a moderate negative correlation between PLR and adverse neonatal outcomes [13]. Maternal SII levels with histological choriomyomatosis correlate with NICU admission rates [14]. Comparatively, mothers of fetuses with ventriculomegaly exhibit higher white blood cell (WBC), and N, M, P, and NLR levels. Multivariate logistic regression analysis suggests that N can serve as a marker for predicting fetal ventriculomegaly. Additionally, ROC curve analysis indicates that maternal WBC and N may possess diagnostic significance in this context [15]. Moreover, higher NLR values in mothers are associated with an increased likelihood of premature births [16,17]. In this study, maternal NLR, MLR, SII, SIRI, and PIV were higher before delivery in the pneumonia group compared to the control group. This suggests that maternal systemic inflammatory indicators before delivery can serve as biomarkers for diagnosing congenital pneumonia.

Early systemic inflammation indicators in neonatal blood hold predictive value for disease occurrence. For instance, preterm neonates typically exhibit significantly higher NLR, PLR, and MLR levels compared to full-term neonates [17]. In neonates with urinary tract infections, the SII before antibiotic treatment can predict renal involvement [18]. Additionally, the NLR shows a negative correlation with neonatal vitamin D levels [19]. Early postnatal NLR, PLR, and SII levels, especially on the first and third days after birth, serve as important predictors of systemic inflammatory response syndrome in term infants [20]. Furthermore, the NLR levels at 72 h after birth may predict BPD in premature infants with intrauterine infections [21]. The NLR in full-term newborns demonstrates high sensitivity and specificity in diagnosing neonatal pneumonia [22]. However, this study did not observe differences in the systemic inflammation indicators between the neonates with congenital pneumonia and those in the control group. This may be attributed to the retrospective nature of our study and the variability in complete blood cell counts within the first 72 h after birth. These early changes could significantly affect the calculated systemic inflammation indicators, potentially explaining the discrepancies between our findings and those of other studies.

This study constructed a prediction model for neonatal pneumonia and grouped neonates based on the intercept value of the prediction model. It was found that neonates with a high predicted probability had longer hospitalization duration, as well as higher antibiotics use and oxygen demand, suggesting that maternal systemic inflammatory indicators before delivery may indicate the severity of the disease. Clinical research has also demonstrated that SIRI correlates with neonatal oxygen demand and the length of ICU stay. In neonates with moderate to severe hypoxic ischemic encephalopathy, neonates exhibit significantly elevated NLR, SII, and SIRI values six hours after birth, with the NLR potentially serving as an independent factor for moderate to severe hypoxic ischemic encephalopathy [23]. Similarly, premature infants born at >32 weeks’ gestational age were classified based on the different oxygen inhalation methods and required oxygen concentrations at 36 weeks of corrected gestational age (PMA) and showed significantly elevated SIRI values. This trend is particularly pronounced in infants with moderate to severe BPD at birth and at 36 weeks of PMA, suggesting that the SIRI could serve as an effective biomarker for predicting moderate to severe BPD [24]. Moreover, children diagnosed with new-onset type 1 diabetes who exhibit higher SIRI values are more likely to experience acute complications and require longer ICU stays. Thus, the SIRI can be used as a predictor of the severity of new-onset type 1 diabetes in children [25].

In conclusion, this study identified the systemic inflammatory indicators in mothers before delivery as predictive markers for congenital pneumonia, and the regression model jointly constructed using the NLR, MLR, SII, SIRI, and PIV before delivery predicted the occurrence of congenital pneumonia better. Additionally, the maternal systemic inflammatory indicators before delivery correlated with the severity of congenital pneumonia in neonates. However, this study is limited by its single-center, retrospective design with a relatively small sample size, which may have introduced confounding factors. Considering amniotic fluid contamination as a pathogenic factor for congenital pneumonia, this study did not include it as a risk factor for evaluation. Future research from our group aims to address these limitations through large-scale, multicenter prospective studies aimed at validating the impact of maternal systemic inflammatory indicators before delivery on congenital pneumonia.

## Figures and Tables

**Table 1:** Comparison of baseline data for the mothers and the neonates between the two groups.

	Observation Group (n = 46)	Control Group (n = 65)	*p*-Value
Birth weight (g) ^a^	3500 (3237.50, 3800.00)	3450 (3100.00, 3700.00)	0.463
Male, n (%)	23 (50.0%)	30 (46.2%)	0.689
Gestational age (weeks) ^b^	39.31 ±1.345	39.11 ± 0.998	0.388
One-minute Apgar score < 7, n (%)	3 (16.1%)	0	0.037 *
Cesarean delivery, n (%)	31 (67.4%)	38 (58.5%)	0.339
Gravidity, n (%)	29 (63.0%)	40 (61.5%)	0.872
Parity, n (%)	26 (56.5%)	35 (53.8%)	0.780
Maternal age (years) ^b^	29.20 ± 4.870	29.35 ± 5.134	0.871
PROM, n (%)	12 (26.1%)	16 (24.6%)	0.860
Nuchal cord, n (%)	22 (47.8%)	22 (33.8%)	0.138
Hypertension, n (%)	7 (15.2%)	12 (18.5%)	0.655
Diabetes mellitus, n (%)	10 (21.7%)	22 (33.8%)	0.165
Anemia, n (%)	5 (10.9%)	8 (12.3%)	0.816

PROM, premature rupture of membranes. ^a^ Median (quartile) [M (P25, P75)], ^b^ mean ± standard deviation. * *p* < 0.05.

**Table 2:** Comparison of systemic inflammatory indicators of the mothers between the two groups before delivery.

	Observation Group (n = 46)	Control Group (n = 65)	*p*-Value
NLR	4.63 (3.89, 6.13)	3.52 (3.05, 3.94)	<0.001 *
PLR	136.83 (99.59, 166.60)	115.82 (99.01, 141.25)	0.066
MLR	0.36 (0.30, 0.44)	0.29 (0.24, 0.35)	<0.001 *
SII	868.79 (734.24, 1221.61)	726.09 (511.84, 897.40)	0.002 *
SIRI	2.47 (1.96, 3.44)	1.76 (1.34, 2.26)	<0.001 *
PIV	474.57 (328.92, 620.58)	368.67 (225.50, 492.14)	0.007 *

NLR, neutrophil-to-lymphocyte ratio; PLR, platelet-to-lymphocyte ratio; MLR, monocyte-to-lymphocyte ratio; SII, systemic immune-inflammation index; SIRI, systemic inflammation response index; PIV, pan-immune-inflammation value. * *p* < 0.05.

**Table 3:** Comparison of systemic inflammatory indicators of the neonates between the two groups.

	Observation Group (n = 46)	Control Group (n = 65)	*p*-Value
Neonatal NLR	3.95 (2.32, 5.05)	3.30 (2.42, 4.39)	0.295
Neonatal PLR	67.31 (44.89, 91.49)	77.49 (60.28, 97.42)	0.109
Neonatal MLR	0.33 (0.20, 0.50)	0.36 (0.22, 0.52)	0.781
Neonatal SII	812.16 (510.60, 1299.07)	894.38 (610.54, 1256.13)	0.570
Neonatal SIRI	4.93 (2.02, 8.13)	3.80 (2.15, 7.07)	0.440
Neonatal PIV	1007.34 (432.21, 2272.05)	981.30 (535.66, 1961.26)	0.905

NLR, neutrophil-to-lymphocyte ratio; PLR, platelet-to-lymphocyte ratio; MLR, monocyte-to-lymphocyte ratio; SII, systemic immune-inflammation index; SIRI, systemic inflammation response index; PIV, pan-immune-inflammation value.

**Table 4:** ROC curves for evaluating the effect of systemic inflammatory indicators of mothers before delivery on the diagnosis of congenital pneumonia in the neonates.

	AUC	95% CI	Cutoff Level	Sensitivity (%)	Specificity (%)	Youden Index
NLR	0.830	0.751–0.909	4.368	63.0	92.3	0.553
MLR	0.727	0.629–0.825	0.360	54.3	86.2	0.405
SII	0.677	0.574–0.780	780.892	67.4	60.0	0.274
SIRI	0.742	0.646–0.839	2.897	43.5	96.9	0.404
PIV	0.651	0.547–0.756	465.034	56.5	69.2	0.257

NLR, neutrophil-to-lymphocyte ratio; MLR, monocyte-to-lymphocyte ratio; SII, systemic immune-inflammation index; SIRI, systemic inflammation response index; PIV, pan-immune-inflammation value.

**Table 5:** Systemic inflammatory indicators of mothers before delivery and the severity of congenital pneumonia.

	Predicted Probability < 0.369 (n = 59)	Predicted Probability > 0.369 (n = 52)	*p*-Value
Hospitalization (days) ^a^	5.00 (4.00, 7.00)	7.00 (5.00, 8.00)	0.009 *
Oxygen inhalation	14 (23.7%)	24 (46.2%)	0.013 *
Antibiotic	16 (27.1%)	42 (80.8%)	<0.001 *

^a^ Median (quartile) [M (P25, P75)]. * *p* < 0.05.

## Data Availability

The data in this article support this study and can be further queried from the corresponding author.
